# Associations between urinary glyphosate and arthritis: an US NHANES analysis

**DOI:** 10.3389/fpubh.2025.1450479

**Published:** 2025-03-13

**Authors:** Xiaoyao He, Liangyu Mi, Miaomiao Zhao, Yuli Ji, Yuting Hu, Yanan Gao, Lixia Qiu, Ke Xu

**Affiliations:** ^1^Department of Statistics, School of Public Health, Shanxi Medical University, Taiyuan, China; ^2^Department of Rheumatology, Shanxi Bethune Hospital, Shanxi Academy of Medical Sciences, Tongji Shanxi Hospital, Third Hospital of Shanxi Medical University, Taiyuan, China

**Keywords:** urinary glyphosate, arthritis, NHANES, cross-sectional study, logistic regression

## Abstract

**Objective:**

As the relationship between urine glyphosate and arthritis in adults in general is still unclear, the study set out to investigate it.

**Methods:**

A total of 1,689 people volunteered in the National Health and Nutrition Examination Surveys (US NHANES). Utilizing a multivariate logistic regression model to explore the association between urine glyphosate concentrations (both continuous with categorical) and the risks of developing arthritis, as well as the risks of various types of arthritis. Non-linear correlations have been investigated using restricted cubic spline and smooth curve fitting. We also conducted additional subgroup analyses using categorical defining features.

**Results:**

Patients with arthritis had urine glyphosate levels of 0.4 ng/mL, while non-arthritic individuals had levels of 0.3 ng/mL (*p* < 0.05). After adjusting for confounding variables, multivariate logistic regression continuous and categorical models demonstrated a significant positive association between elevated urinary glyphosate levels and arthritis risk [1.2 (1.0, 1.4)]. This association was observed in the osteoarthritis (OA) subgroup, with an odds ratio of 1.3 (95% CI: 1.1, 1.6), but was not found in the rheumatoid arthritis (RA) or other arthritis subgroups. Smooth curve fitting and RCS regression analyses further elucidate that urine glyphosate levels exhibit a dose-dependent relationship with the risks of both arthritis and OA, adhering to a linear trend (with a *p*-value for nonlinearity exceeding 0.05). Subsequent subgroup studies showed that in certain groups of people, the positive relationship between urine glyphosate and arthritis remained significant.

**Conclusion:**

Increased exposure to urine glyphosate may be associated with an elevated risk of arthritis, particularly in the subgroup of osteoarthritis.

## 1 Introduction

An acute or chronic condition that affects one or more joints is called arthritis. Its clinical spectrum may include stiffness, discomfort, and joint deformity as well as edema (1). Both of the predominant types of arthritis are osteoarthritis (OA) and rheumatoid arthritis (RA) (2, 3). Arthritis patients experience a reduced quality of life compared to individuals with gastrointestinal, respiratory, or cardiovascular conditions, and it ranks as the primary cause of disability among Americans (4, 5). Furthermore, the disease imposes a substantial economic and healthcare burden on the nation (2, 6). By 2040, there is expected to be a notable rise in the amount of persons in the US who receive a diagnosis of arthritis from their doctors (7). With obesity, smoking, food choices, and hormone use as risk factors, it is readily apparent that arthritis remains an important challenge in clinical and public health systems. Additionally, it makes sense to actively pursue other risk factors for the disease’s development in order to set the stage for the widespread implementation of successfully implemented public health interventions (8, 9).

A popular herbicide in residential, commercial, and agricultural contexts is glyphosate. Since their initial release in 1974, glyphosate-based herbicides have grown in favor both domestically and abroad. They frequently contaminate the air, precipitation, and drinking water sources (10, 11). Urine contains glyphosate because it enters the body by inhalation and skin contact and is largely not metabolized there (12). According to the most recent cohort study, individuals who ate wholemeal bread had greater urine glyphosate contents, which may indicate that glyphosate can also enter the body from food (13). Numerous relevant studies have shown that contacting glyphosate increases the incidence of non-Hodgkin’s cancer of the lymph no casting doubt on a medication’s safety for use as a pesticide (14), is linked to a higher risk of diabetes (15), and may be linked to cognitive impairment in older persons (16). besides glyphosate exposure was linked to abnormalities in hearing, depression, anemia, hypertension, obesity, and cognitive function (17–19).

According to a cohort research (20), pesticides containing glyphosate raise the incidence of rheumatoid arthritis. Interestingly, recent research has suggested that effectively preventing OA in environments with glyphosate exposure can be achieved by adjusting leisure time physical activity and body mass index types (21). Though the precise mechanism by which glyphosate affects the immune system is unknown, exposure to glyphosate-enriched air samples and breathing in small amounts of this chemical alone increased mast cell degranulation of fat eosinophil and white blood cells counts, and the manufacturing of TSLP, IL-13, IL-5, along with IL-33, which in turn induced inflammation in the lung dependent on IL-13 and promoted Th2 type cytokines (22). It is noteworthy that glyphosate has a direct impact on the differentiation of Th cells, resulting in an imbalance between Th1/Th2 specific populations and promoting an elevated Th2 response (23). Research indicates that glyphosate also causes oxidative stress in animal products gastrointestinal epithelial cells, increases the amount of innate immune factors, and triggers autophagy through the Nrf2/HO-1 pathway. Additionally, excessive exposure to herbicides containing glyphosate may result in cytotoxicity through these mechanisms (24). In addition to causing experimental arthritis to worsen, oxidative stress can cause arthritis (25, 26). Furthermore, human health may be negatively impacted by glyphosate herbicide through endocrine pathways (27). Exposure to glyphosate enhanced PTEN expression and activated the cellular apoptotic mechanism in hepatic L8824 cells, resulting in the production of apoptotic cells (28). In summary, glyphosate has diverse methods of action *in vivo*; nevertheless, its precise means of action in connection with arthritis in humans remains unknown.

The current investigation indicates that no assessment has been done on the connection between urinary glyphosate and arthritis. This study aims to explore whether urinary glyphosate is associated with arthritis. The data utilized in this cross-sectional investigation of a representative sample of the US population is based on the National Health and Nutrition Examination Survey (US NHANES), which was carried out between 2013 and 2016.

## 2 Methods

### 2.1 Study population

**Figure 1:**
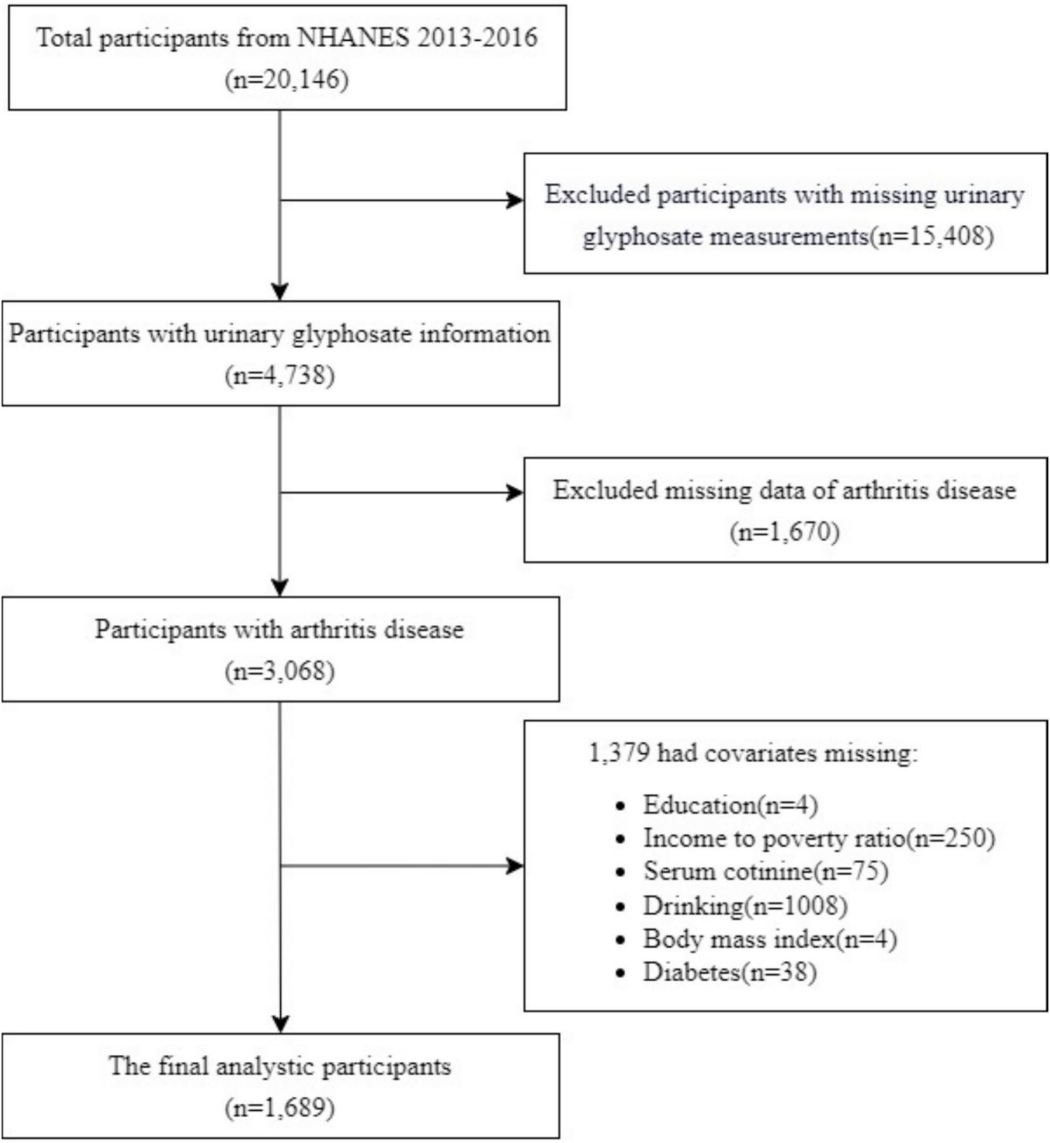
The individual’s choice procedure flow chart.

The National Health and Nutrition Examination Survey (US NHANES)^1^ was used to recruit individuals for the study. Because urine glyphosate data was only available for two US NHANES cycles (2013–2014 and 2015–2016), the surveys from those two cycles were included in the current analysis. The selection procedure for our study is shown in Figure 1. Measurements of glyphosate were taken from one-third of the participants who agreed to have their lab samples analyzed in the future. We screened for missing data on urinary glyphosate (*n* = 15,408) and arthritis (*n* = 1,670) among 20,146 individuals. Furthermore, we excluded data lacking relevant covariates (*n* = 1,379). Finally, 1,689 subjects with complete data were included in our current analysis.

### 2.2 Exposure and outcomes

Utilizing two-dimensional offline ion chromatography in conjunction with mass spectrometry (IC-MS/MS) and 200 microliters of urine, a laboratory technique was used to extract glyphosate from the urine. For the urinary glyphosate focused attention, an inferior restrict of discovery (LLOD) within 0.2 ng/mL was reported, which was reported as ng/ml. Values underneath LLOD will be approximated using the product of the square roots of two times the LLOD value under the US NHANES program.

Arthritis was characterized as self-identified arthritis for the objective of this study as follows: “Have you ever received the diagnosis of arthritis from a physician or other health care provider?” and “Which type of arthritis was it?” The individuals who responded “yes” participated in the study and were classified for arthritis based on the second question.

### 2.3 Covariates

Based on previous research on the interaction between environmental pollutant exposure and arthritis (21, 29–31), the covariates selected for this study mainly include the following: categorical variables included gender, age, ethnicity, education, family poverty-income ratio (PIR), marital status, drinking, body mass index (BMI), diabetes, and hypertension. Continuous covariates included serum cotinine (ng/mL), which has been identified as a potent biomarker to indicate cigarette exposure (32). An anthropometric covariate called body mass index (BMI, kg/m^2^) is calculated by dividing weight (kg) by squared height (m). People who are classified as having diabetes and hypertension are those who have received a diagnosis from a medical professional (31).

### 2.4 Statistical analysis

The subjects were divided into two groups according to the presence or absence of arthritis. To evaluate variations in the baseline variables, the student *t*-test (continuous variables) and the chi-square test (categorical variables) were employed. Logistic regression models were employed to investigate the relationship between glyphosate and arthritis. Model 1 had uncorrected covariates; Model 2 adjusted for gender, age, race, education, PIR, and marital status. Model 3 adjusted for gender, age, race, education, PIR, marital status, BMI, serum cotinine, drinking, diabetes and hypertension. In the model, urinary glyphosate has been produced in both a constant and categorized variable, with the lowest quartile serving as the reference group. We employed a Logistic regression model to investigate the dose–response relationship between ln-transformed glyphosate levels in urine. Smoothed curves and threshold effects analysis were employed to examine the relationship between ln-converted glyphosate levels in urine and arthritis. Additionally, we applied Restricted Cubic Splines (RCS) regression with three knots (at the 10th, 50th, and 90th percentiles) to explore the nonlinear association between ln-transformed glyphosate in urine and arthritis, as well as its subtypes. Using a stratified multivariate logistic regression model, subgroup analyses were carried out for arthritis risk and age, as well as for sex, race, education level, income-poverty ratio, marital status, BMI, diabetes and hypertension. Additionally, stratified factors were considered as possible moderators of effects. Including an interaction term and applying likelihood ratio tests to assess heterogeneity. EmpowerStats was utilized to examine the data, and R version 4.3.3 was employed for all analyses. Every threshold for statistical significance was set at *p* ≤ 0.05 (bilateral).

## 3 Results

### 3.1 Baseline characteristics

The study included 1,689 eligible people, 410 of whom were diagnosed with arthritis [OA 190 (12.9%), RA 68 (5.0%), Other 152 (10.6%)], and 1,279 of whom served as controls (Table 1). Significant variations were seen when arthritis was present in the following demographics: gender, age, race, marital status, BMI, diabetes and hypertension. Patients with arthritis are not only more likely to be older, female, non-Hispanic White, married, and obese, but they also have greater rates of diabetes and hypertension. Additionally, there was a difference that was significantly different (*p* < 0.001) among the glyphosate levels in urine and arthritis.

**Table 1:** Traits connected to the research population based on the presence of arthritis.

Characteristics	Total (*n* = 1,689)	Non- arthritis (*n* = 1,279)	Arthritis (*n* = 410)	*p*-value
Arthritis Category, *n* (%)				
OA	1,689	1,499(88.7)	190(11.3)	
RA	1,689	1,621(96.0)	68(4.0)	
Other	1,689	1,537(91.0)	152(10.6)	
Glyphosate ng/mL	0.3 (0.1–8.2)	0.3 (0.1–4.0)	0.4 (0.1–8.2)	<0.001
Gender, *n* (%)				<0.001
Male	877 (51.9)	698 (54.6)	179 (43.7)	
Female	812 (48.1)	581 (45.4)	231 (56.3)	
Age, *n* (%)				<0.001
20–34	493 (29.2)	469 (36.7)	24 (5.9)	
35–49	481 (28.5)	402 (31.4)	79 (19.3)	
≥50	715 (42.3)	408 (31.9)	307 (74.9)	
Race, *n* (%)				<0.001
Mexican American	236 (14.0)	189 (14.8)	47 (11.5)	
Non-Hispanic white	774 (45.8)	547 (42.8)	227 (55.4)	
Non-Hispanic black	301 (17.8)	231 (18.1)	70 (17.1)	
Other	378 (22.4)	312 (24.4)	66 (16.1)	
Education, *n* (%)				0.148
Less than 9th grade	82 (4.9)	62 (4.8)	20 (4.9)	
9–11th grade	165 (9.8)	130 (10.2)	35 (8.5)	
High school graduate/GED /equivalent	378 (22.4)	288 (22.5)	90 (22.0)	
Some college/AA degree	584 (34.6)	422 (33.0)	162 (39.5)	
College graduate/above	480 (28.4)	377 (29.5)	103 (25.1)	
PIR, *n* (%)				0.997
<1	302 (17.9)	229 (17.9)	73 (17.8)	
1–4.99	1,015 (60.1)	768 (60.0)	247 (60.2)	
≥5	372 (22.0)	282 (22.0)	90 (22.0)	
Marital status, *n* (%)				<0.001
Married	873 (51.7)	661 (51.7)	212 (51.7)	
Never married	343 (20.3)	297 (23.2)	46 (11.2)	
Other	473 (28.0)	321 (25.1)	152 (37.1)	
Serum cotinine ng/mL	61.9 ± 124.9	62.1 ± 126.9	61.4 ± 118.6	0.923
Drinking, *n* (%)				0.860
Drinker	39 (2.3)	30 (2.3)	9 (2.2)	
Non-drinker	1,650 (97.7)	1,249 (97.7)	401 (97.8)	
BMI, *n* (%)				<0.001
<25	510 (30.2)	423 (33.0)	87 (21.2)	
25–29.99	536 (31.7)	414 (32.4)	122 (29.8)	
≥30	643 (38.1)	442 (34.6)	201 (49.0)	
Diabetes, *n* (%)				<0.001
Yes	168 (9.9)	98 (7.7)	70 (17.1)	
No	1,521 (90.1)	1,181 (92.3)	340 (82.9)	
Hypertension, *n* (%)				<0.001
Yes	568 (33.6)	331 (25.9)	237 (57.8)	
No	1,121 (66.4)	948 (74.1)	173 (42.2)	

Continuous variables that followed a normal distribution were represented as mean ± SD; continuous variables that did not follow a normal distribution were represented as median (IQR); categorical variables were represented as *n* (%).

### 3.2 Urinary glyphosate and arthritis risk are associated

**Figure 2:**
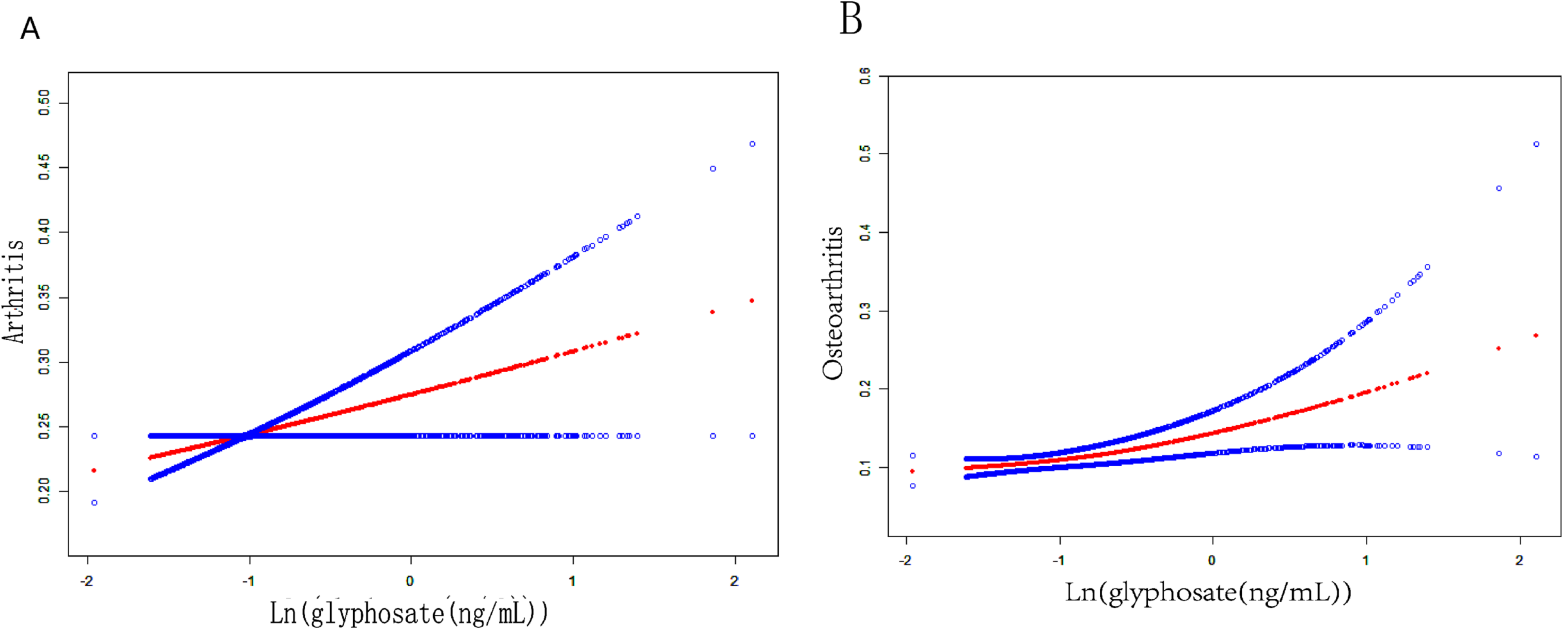
Fitting smooth curves for the correlation between log-transformed concentrations of glyphosate and arthritis, including its subtypes: **(A)** arthritis, **(B)** OA.

**Figure 3:**
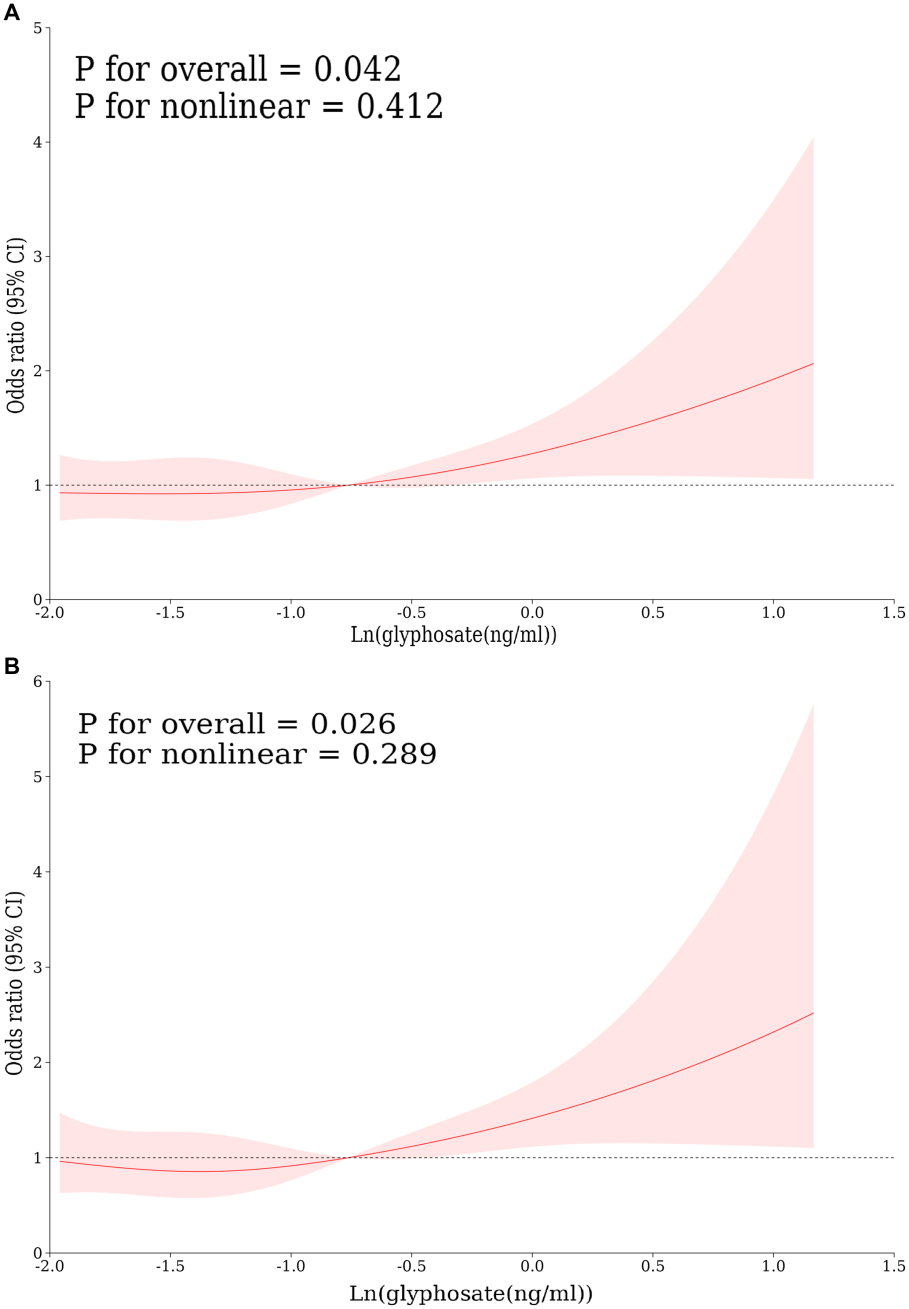
Restricted cubic spline curves for the correlation between log-transformed concentrations of glyphosate and arthritis, including its subtypes: **(A)** arthritis, **(B)** OA, adjusted according to model 3.

This study developed three models to examine a connection concerning urine glyphosate alongside arthritis (Table 2). Urinary glyphosate was found to be substantially linked to a higher incidence of arthritis in fully corrected continuous models (OR 1.2, 95% CI 1.0–1.4, *p* = 0.049). The results of the categorical models indicated that the subjects in the upper quartiles of Model 1 (OR 1.6, 95% CI 1.2–2.1), Model 2 (OR 1.6, 95% CI 1.1–2.2), and fully adjusted Model 3 (OR 1.5, 95% CI 1.1–2.1) had a statistically significant higher likelihood for arthritis in comparison to the bottom reference ranking. Importantly, the correlation was significant for the arthritis subtype OA (OR 1.3, 95% CI 1.1–1.6, *p* = 0.011). However, there was no apparent association between glyphosate and RA. To further assess the linear relationship between glyphosate and the risk of arthritis and its subtype (OA), we employed smoothed curve fitting (Figure 2) and RCS modeling (Figure 3). Additionally, Figure 2 suggests a nonlinear trend for OA at lower concentrations, prompting further exploration of threshold effects (Table 3). Unfortunately, no significant inflection point was observed (log-likelihood ratio > 0.05). This analysis revealed a linear correlation between glyphosate and the risk of arthritis and its subtype (OA) (all *p*-values for nonlinearity >0.05). Notably, the dose–response relationship was also significant (overall *p* < 0.05).

**Table 2:** The risk of arthritis and urine levels of glyphosate are associated by multiple logistic regression analyses.

Outcomes	Continuous models		Categorical models				
	Ln OR (95%CI)	*P*-value	Q1	Q2	Q3	Q4	*P* for trend
				OR (95% CI)	OR (95% CI)	OR (95% CI)	
Arthritis							
Model 1	**1.3 (1.1, 1.4)**	0.002	Ref	1.1 (0.8, 1.5)	1.2 (0.9, 1.6)	**1.6 (1.2, 2.1)**	0.002
Model 2	**1.2 (1.0, 1.4)**	0.025	Ref	1.2 (0.9, 1.7)	1.2 (0.9, 1.7)	**1.6 (1.1, 2.2)**	0.009
Model 3	**1.2 (1.0, 1.4)**	0.049	Ref	1.2 (0.8, 1.7)	1.1 (0.8, 1.6)	**1.5 (1.1, 2.1)**	0.017
OA							
Model 1	**1.4 (1.1, 1.7)**	<0.001	Ref	0.9 (0.6, 1.4)	1.0 (0.6, 1.5)	**1.9 (1.3, 2.8)**	<0.001
Model 2	**1.3 (1.1, 1.6)**	0.009	Ref	0.9 (0.6, 1.5)	1.0 (0.6, 1.6)	**1.9 (1.2, 2.8)**	0.003
Model 3	**1.3 (1.1, 1.6)**	0.011	Ref	1.0 (0.6, 1.5)	0.9 (0.6, 1.5)	**1.8 (1.2, 2.8)**	0.004
RA							
Model 1	0.9 (0.7, 1.3)	0.727	Ref	0.9 (0.4, 1.7)	1.0 (0.5, 1.9)	0.7 (0.3, 1.4)	0.257
Model 2	0.9 (0.7, 1.3)	0.585	Ref	0.9 (0.4, 1.7)	1.0 (0.5, 2.0)	0.6 (0.3, 1.3)	0.207
Model 3	0.9 (0.6, 1.2)	0.514	Ref	0.8 (0.4, 1.7)	0.9 (0.5, 1.8)	0.6 (0.3, 1.3)	0.175
Other							
Model 1	1.1 (0.8, 1.5)	0.664	Ref	1.6 (1.0, 2.5)	1.5 (0.9, 2.3)	1.5 (0.9, 2.3)	0.105
Model 2	1.0 (0.7, 1.5)	0.822	Ref	**1.6 (1.0, 2.6)**	1.5 (0.9, 2.4)	1.4 (0.9, 2.2)	0.19
Model 3	1.0 (0.7, 1.4)	0.985	Ref	1.6 (1.0, 2.5)	1.4 (0.9, 2.3)	1.3 (0.8, 2.1)	0.276

Model 1 had no adjustments made. Model 2 adjusted for gender, age, race, education, PIR, and marital status. Model 3 adjusted for gender, age, race, education, PIR, marital status, BMI, serum cotinine, drinking habits, diabetes, and hypertension. Bold: *p* < 0.05.

**Table 3:** Threshold effect analysis of urinary glyphosate in OA using a two-piecewise linear regression model.

Outcome	Periodontitis	
	OR (95% CI)	*P*-value
Fitting by weighted linear regression mode	1.30 (1.06, 1.60)	0.0113
Fitting by the weighted two-piecewise linear regression model		
Inflection point	−0.78	
Ln(glyphosate) < −0.78	0.98 (0.65, 1.47)	0.9197
Ln(glyphosate) ≥ −0.78	1.71 (1.16, 2.53)	0.0072
Log-likelihood ratio test	0.114	

### 3.3 Subgroup analysis

**Figure 4:**
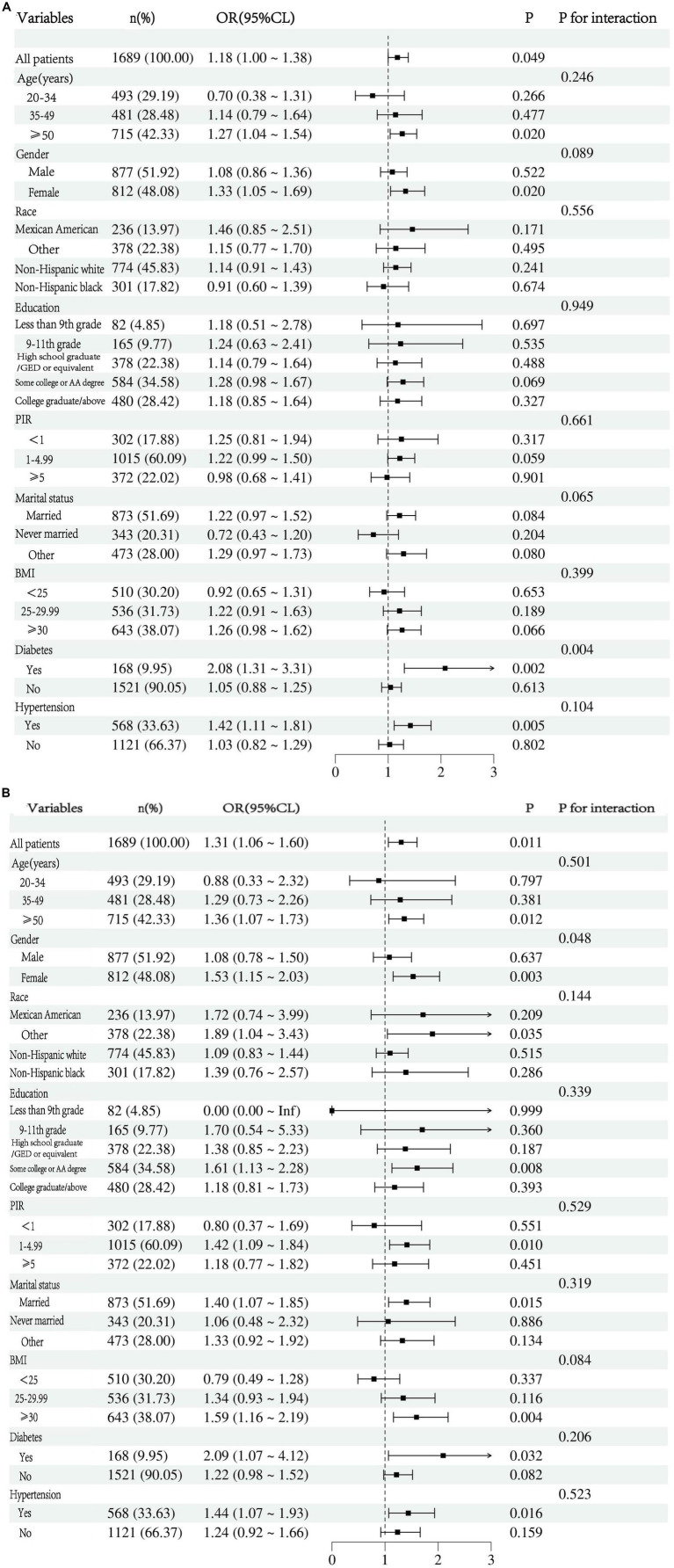
**(A)** Subgroup analysis for the correlation between glyphosate exposure and arthritis risk. **(B)** Subgroup analysis for the correlation between glyphosate exposure and OA risk.

This study employed subgroup analysis and interaction tests, stratified by age, sex, ethnicity, education level, income-to-poverty ratio, marital status, BMI, diabetes, and hypertension, to identify potentially distinct population subsets and to ascertain whether the association between urine glyphosate and arthritis was consistent within the broader community. The data presented in Figure 4A indicates a significant positive correlation between the following subgroups: age subgroup of 50 years or older (OR 1.27, 95%CI 1.05–1.54, *p* = 0.020), Females (OR 1.33, 95%CI 1.05–1.69, p = 0.020), diabetes (OR 2.08, 95%CI 1.31–3.31, *p* = 0.002), and hypertension (OR 1.42, 95%CI 1.11–1.81, *p* = 0.005) were significantly positively correlated. The interaction analysis revealed that diabetes has a significant modifying effect (p for interaction =0.004). The subgroup and interaction analysis between glyphosate and OA shown in Figure 4B indicates that the following factors were significantly positively correlated: Age subgroup of 50 years or older (OR 1.36, 95% CI 1.07–1.73, *p* = 0.012), Females (OR 1.53, 95% CI 1.15–2.03, *p* = 0.003), Other races (OR 1.89, 95% CI 1.04–3.43, *p* = 0.035), Some college/AA degree (OR 1.61, 95% CI 1.13–2.28, *p* = 0.008), PIR subgroup of 1–4.99 (OR 1.42, 95% CI 1.09–1.84, *p* = 0.010), Married (OR 1.40, 95% CI 1.07–1.85, *p* = 0.015), BMI ≥ 30 (OR 1.59, 95% CI 1.16–2.19, *p* = 0.004), diabetes (OR 2.09, 95% CI 1.07–4.12, *p* = 0.032), and hypertension (OR 1.44, 95% CI 1.07–1.93, *p* = 0.016). The interaction analysis further revealed that gender has a significant modifying effect (p for interactio*n* = 0.048).

## 4 Discussion

The purpose of this study was to assess the relationship between glyphosate in urine and arthritis (including its subtypes) in the US population. According to a cross-sectional investigation involving 1,689 subjects, the current investigator found a significant correlation between urine glyphosate levels and the severity of arthritis, specifically its subtype OA. Even after controlling for other variables, this association remained, indicating that urine glyphosate may be an early warning sign for the onset of arthritis. In our study, statistical adjustment for multiple comparisons was not performed in the subgroup analysis, which may increase the risk of Type I error. However, considering the exploratory nature of the analysis and the constraints of sample size, we chose to retain the unadjusted *p*-values to balance statistical power and false positive control. This practice has precedence in similar environmental epidemiological studies (31, 33). If the Bonferroni correction were applied (adjusted *α* = 0.017), the significance in the OA subgroup would no longer hold, suggesting potential inadequacy in the robustness of the current results. Therefore, we recommend treating the OA-related findings as preliminary and suggest further validation in independent cohorts.

Glyphosate is a widely used and increasingly ubiquitous component in pesticides. The International Agency for Research on Cancer (IARC) designated glyphosate as a possible humanity carcinogenesis (Group 2A) in 2015 (34). A large prospective cohort analysis conducted in 2018 revealed that glyphosate did not significantly correlate with either solid tumor or lymphoid malignancy (35). With its complete prohibition of glyphosate in place since 2015, Sri Lanka is the first nation around the globe to have done so. This prohibition was eventually fully abolished in 2022, however it was only in place for a brief time as it was substantially lifted in 2018 (36). Because of this, the topic of whether glyphosate is dangerous remains unresolved, but there are legitimate concerns over the chemical’s effectiveness as a herbicide (37). It is worth noting that urinary glyphosate concentrations do not exhibit bioaccumulation (38), and in the NHANES database, glyphosate concentrations in urine are measured only once per cycle for each participant. Therefore, the measured concentrations may not accurately reflect long-term exposure or explain variations in individual exposure over time.

Soil bacteria metabolize glyphosate to create aminomethyl phosphate (AMPA). Human gut microbial metabolism is suggested by the post-poisoning blood AMPA assay. *In vitro* and in rats, glyphosate, glyphosate formulations, and AMPA induce oxidative stress (34). There may be a beneficial association between glyphosate consumption and several biological markers of oxidative stress in the urine, according to the 2023 cohort study, which examined the relationship among glyphosate exposure and oxidative stress signatures in the urine (39). Although the observed difference in urine glyphosate concentrations (0.3 vs. 0.4 ng/mL) in this study was statistically significant, the toxicological significance of this absolute difference remains unclear. The median urine glyphosate concentration is generally consistent with levels reported in previous studies (0.392 ng/mL) (40), and this slight difference may stem from the sensitivity of large sample sizes rather than a definitive dose–response relationship. Regarding the question of at what concentration urine glyphosate may produce toxicological effects, there is currently no established threshold. However, some studies and data have provided clues about the relationship between glyphosate and human health. A cohort research revealed a dose–response connection between wholemeal bread consumption and increased urine glyphosate concentrations (13). This finding could suggest that other crops that have been eaten have been impacted by the usage of the herbicide glyphosate. Higher exposure to glyphosate was linked to poorer cognitive performance scores, increased risk of serious depressive symptoms, and severe hearing impairments (17). Blood testosterone concentrations are impacted by a dose-dependent rise in urine glyphosate concentration (41). Elevated exposure levels are linked to a higher chance of anemia (18). Obesity, cardiovascular disease, hypertension, and type 2 diabetes are more common in people with greater glyphosate levels (19). For this reason, it is crucial for human health to control the intake of glyphosate and to lessen any negative impacts it may have.

Although there is limited information regarding the link between glyphosate and arthritis, studies have shown that elevated urinary glyphosate concentrations are associated with an increased likelihood of OA, a relationship that is modulated by leisure-time physical activity and body mass index. Among individuals with high BMI, the correlation between glyphosate and OA is more significant (21). The subgroup analysis results of the current study indicate a significant positive correlation between glyphosate and OA for the subgroup with BMI ≥ 30 (OR 1.59, 95% CI 1.16–2.19, *p* = 0.004), which aligns with the expectations of previous research. A cohort study indicates that glyphosate is one of the 15 chemicals that may increase the risk of RA in women when exposed through pesticide application in agriculture, solvents, and fertilizers. Compared to not using any pesticide at all, using glyphosate was associated with an increased incidence of rheumatoid arthritis (OR = 1.4; 95% CI 1.0, 2.1) (20). However, the multivariate logistic regression analysis conducted in this study revealed no significant correlation between glyphosate and RA. The possible reason for this outcome may be the relatively small sample size of RA data included in the study. Reactive oxygen species (ROS) and nitrogen species that react (RNS), produced by phagocytes and essential for the removal of germs in healthy individuals, are overproduced in arthritic patients due to oxidative stress. ROS are eliminated by antioxidant enzymes and low molecular mass compounds that are antioxidants. On the other hand, ROS generation surpasses antioxidant defense, leading to oxidative stress (25).

The synthesis of autoantibodies and the creation of novel epitopes in arthritis are caused by oxidative stress-induced oxidative post-translational modifications. Type II collagen is one example of a protein antigen that has undergone chemical alteration that can cause or worsen experimental arthritis (26). Sex hormones manage the immunoinflammatory response; physiological concentrations of androgens have been shown to be anti-inflammatory, while physiological number of female hormones are known to enhance the immune response, or at least humoral immunity. The higher the urinary glyphosate quantity, the more likely it is that the blood concentration of sex hormones will be (41). Estrogen levels are increased in inflammatory areas, such as synovial fluid. Interesting but complicated is the clinical application of sex hormones as supplementary therapy in rheumatoid arthritis. There is ongoing debate concerning interindividual atmospheric and metabolic changes in hormonal status (42). Ultimately, glyphosate may potentially impact endocrine pathways, sex hormones, or oxidative stress related to arthritis, though direct evidence linking glyphosate to human arthritis through these mechanisms is currently lacking. Future experimental studies are needed to support these hypotheses. Consequently, it makes sense that the current study’s conclusion that glyphosate consumption may raise the incidence of arthritis given the detrimental impact of glyphosate on persons. Additional pharmacological and experimental investigations are required to comprehend the mechanisms underlying glyphosate’s effects on human arthritis.

Based to the US NHANES database, this research offers the first significant epidemiological evidence connecting urinary glyphosate concentration with arthritis as it is the first to investigate the relationship among urinary glyphosate concentration and arthritis. However, the findings regarding glyphosate exposure patterns and associated health effects derived from the analysis of the unique US NHANES data may primarily reflect characteristics of the American population. Given the variability in glyphosate exposure patterns globally, encompassing differences in agricultural practices, environmental regulations, and demographic characteristics, the results of this study should be interpreted with caution and should not be overgeneralized to other countries or regions. Future research needs to be conducted on a global scale to comprehensively assess the diversity of glyphosate exposure and its health impacts. The study has a number of drawbacks. Firstly, there may be a certain degree of selection bias in the sample, potentially including only individuals who are more easily accessible or more willing to participate in the survey. Secondly, the small sample size in the RA subgroup analysis may result in false-negative outcomes. Thirdly, the consideration of confounding factors may not be comprehensive (for example, dietary patterns, occupational exposure to pesticides, and the use of NSAIDs). Fourthly, this is a cross-sectional study, which cannot establish causal relationships. To elucidate causality, large-scale prospective studies are needed.

## 5 Conclusion

After adjusting for various covariates, the study found a significant positive correlation between glyphosate and arthritis, with a notable positive association also observed in the OA subtype. However, no such link was identified in RA or other types of arthritis. RCS analysis indicated a significant, potential linear correlation between glyphosate and arthritis, as well as its subtype OA. Subgroup analysis revealed that exposure to glyphosate may increase the likelihood of developing arthritis and its OA subtype among older individuals, females, and those with diabetes and hypertension. Interaction analysis further demonstrated that factors such as diabetes and gender significantly modified this association. While more research is necessary to determine a sense of direction and clinical implications of these relationships, the current study’s findings highlight the importance of continuing to investigate how exposure to glyphosate affects adults with arthritis.

## Data Availability

The datasets presented in this study can be found in online repositories. The names of the repository/repositories and accession number(s) can be found at: https://www.cdc.gov/nchs/nhanes.

## Footnotes

^1^ https://www.cdc.gov/nchs/nhanes
